# A Rare Case of Double-System With Ectopic Ureteral Openings Into Vagina

**DOI:** 10.3389/fped.2018.00176

**Published:** 2018-06-19

**Authors:** Carmen Duicu, Eva Kiss, Iunius Simu, Cornel Aldea

**Affiliations:** ^1^1st Department of Paediatrics, University of Medicine and Pharmacy, Târgu Mureş, Romania; ^2^Radiology Department, University of Medicine and Pharmacy, Târgu Mureş, Romania; ^3^Pediatric Nephrology Department, Emergency Clinical Hospital for Children Cluj-Napoca, Cluj-Napoca, Romania

**Keywords:** urinary incontinence, ectopic ureter, vagina, computed tomography, child

## Abstract

The presence of an ectopic ureter may be indicated by continuous wetting, despite a normal voiding pattern, especially in girls. In most cases, an ectopic ureter is associated with a duplex collecting system and complete ureteral duplication. A 5-year-old girl presented with urinary incontinence regardless of the successful toilet training and a suspicion of left duplex kidney on a previous ultrasound. Contrast-enhanced computed tomography revealed a double left kidney with double ureters, both inserting together into the vagina. The surgical treatment consisted in the “en block” reimplantation of the ectopic ureters into the bladder, with complete resolution of the symptoms. The reported case does not represent just a typical presentation of a single ectopic ureter, as the duplex kidney system had *ectopic both ipsilateral ureters* (with insertion into the vagina). This case reminds us that congenital abnormalities of the genito-urinary tract should be considered in case of urinary incontinence and recurrent urinary tract infections.

## Introduction

A constant urinary leakage is a common problem during childhood, but generally there is no underlying abnormality of the urinary tract. Urinary incontinence is the main symptom of an ectopic ureter, especially in females, while in both sexes, an ectopic ureter may present as a prenatal diagnosis because of a urinary tract infection (UTI), or congenital obstructive uropathy (1). These two risk factors can finally lead to chronic renal failure. The diagnosis is usually made during childhood because of recurrent UTIs or urinary incontinence.

In most cases, the ectopic ureter originates from the upper renal pole of the duplex-system (80%) while single-system ureter ectopia is exceptional (20%). Compared to the ectopic ureter of the duplicated-system, diagnosis of the single-system ectopia can be delayed because it usually drains small dysplastic and hypofunctional kidneys, which may be difficult to identify with conventional imaging, like intravenous urography and urethrocystoscopy (2). Our child is a very rare case of double ureteral ectopia presenting with continuous urinary dribbling and this report highlights the challenges in the diagnosis and treatment of this rare condition.

Evaluation and management of young patients who present with urinary incontinence after toilet training or recurrent UTIs can often be difficult. Voiding dysfunction, overactive bladder, as well as ectopic ureter are some disorders that can manifest with these symptoms (3).

## Case report

A 5-year-old girl was referred to our hospital for the investigation of urinary incontinence. The patient had continuous low volume urine leakage requiring 4–5 daily pads. The parents could not specify whether there was any connection with standing, coughing, or effort and she had no urge to void.

She was constantly wet but had normal voiding habits. On initial physical examination the external genitalia appeared normal with no vaginal pooling of urine or ectopic ureteral orifice. However, a long-term external genitalia examination revealed normal urethral and vaginal openings, with an intermittent urine leakage through the vaginal orifice, which slightly increased in abdominal pressure.

In her past history she had recurrent febrile urinary tract infections (UTIs) since her infancy. At the age of 3 she underwent an abdominal ultrasound that suspected a double left kidney. A voiding cystourethrogram (VCUG) was performed and a vesicoureteral reflux (VUR) grade III on the right kidney was found. She was given chronic chemoprophylaxis without any UTI recurrence. She had a good toilet training and gave up daytime diapers around the age of 3. At this moment parents noticed the urinary incontinence, but this didn't bother them. At the age of 4 parents asked a urologist for help, who considered the symptoms to be the result of an overactive bladder and anticholinergic treatment was recommended. No improvement was noticed, and for this reason the little girl was sent to our department for further investigation.

Complete blood count, biochemical tests, and urinalysis were all normal and the urine culture was negative.

**Figure 1:**
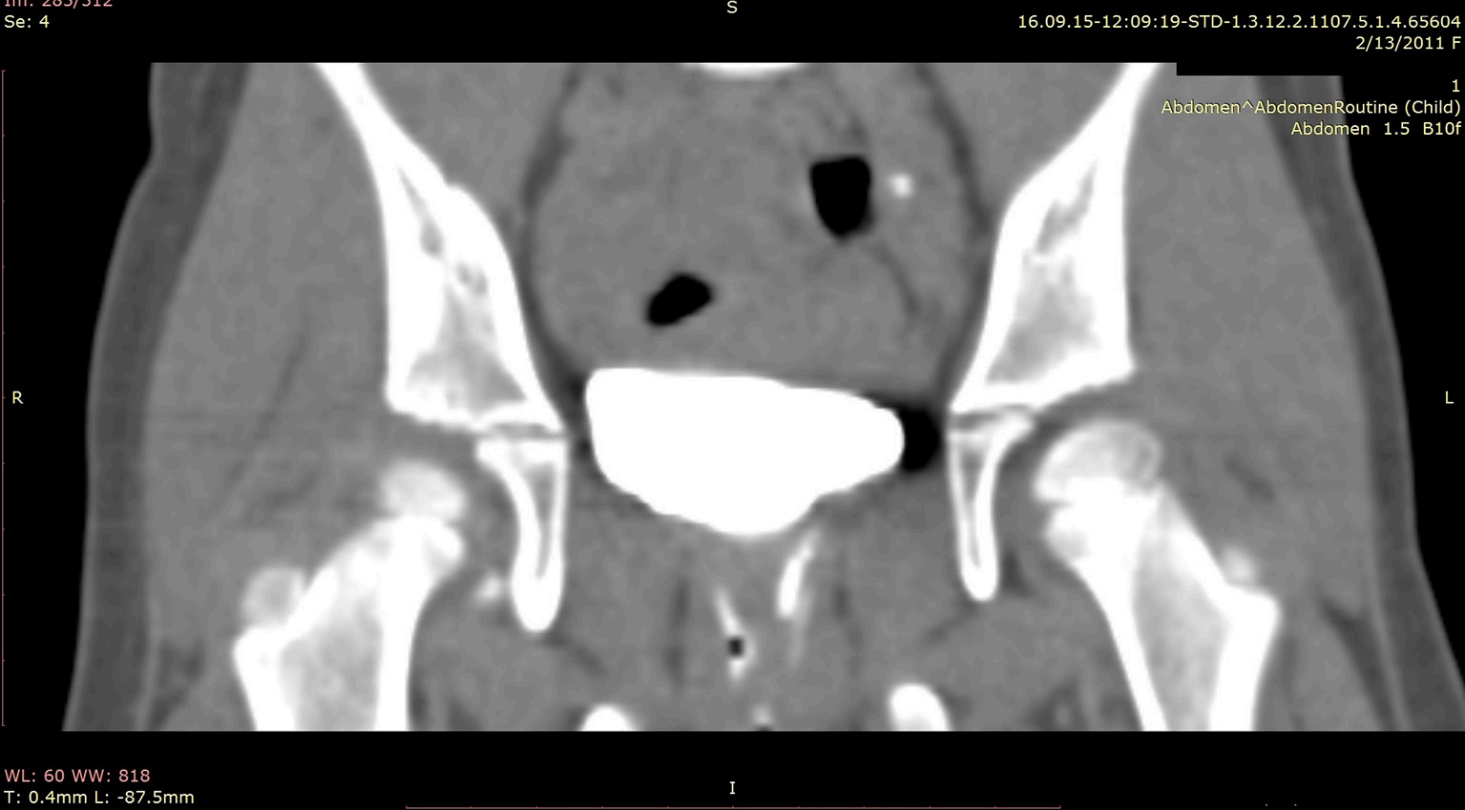
CT urography coronal view—double ectopic left ureter abuts the vagina under the bladder.

**Figure 2:**
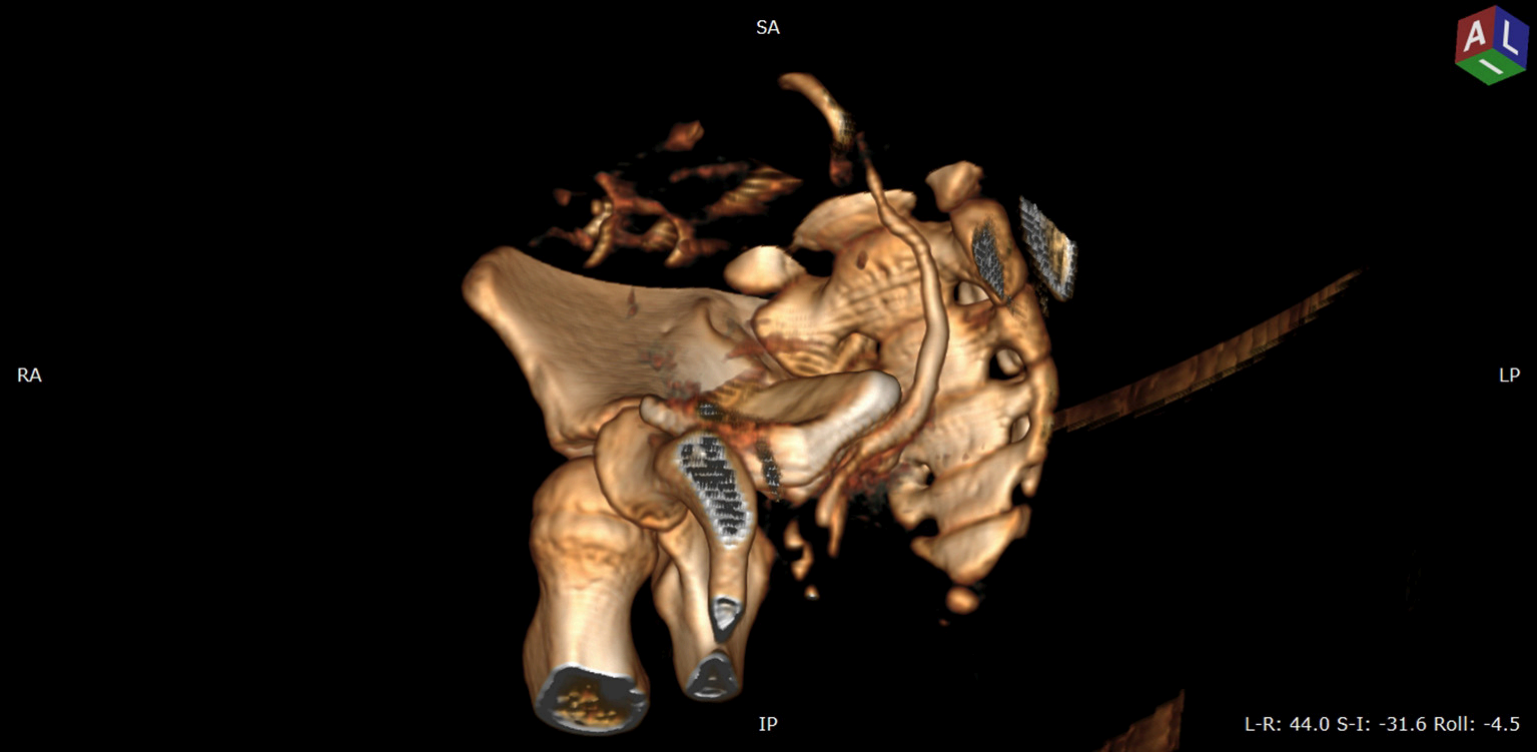
VRT (volume rendering technique) CT image, urographic sequence—oblique anterior view after removing left coxal bone—paravesical path of left ureter that insert into vagina.

**Figure 3:**
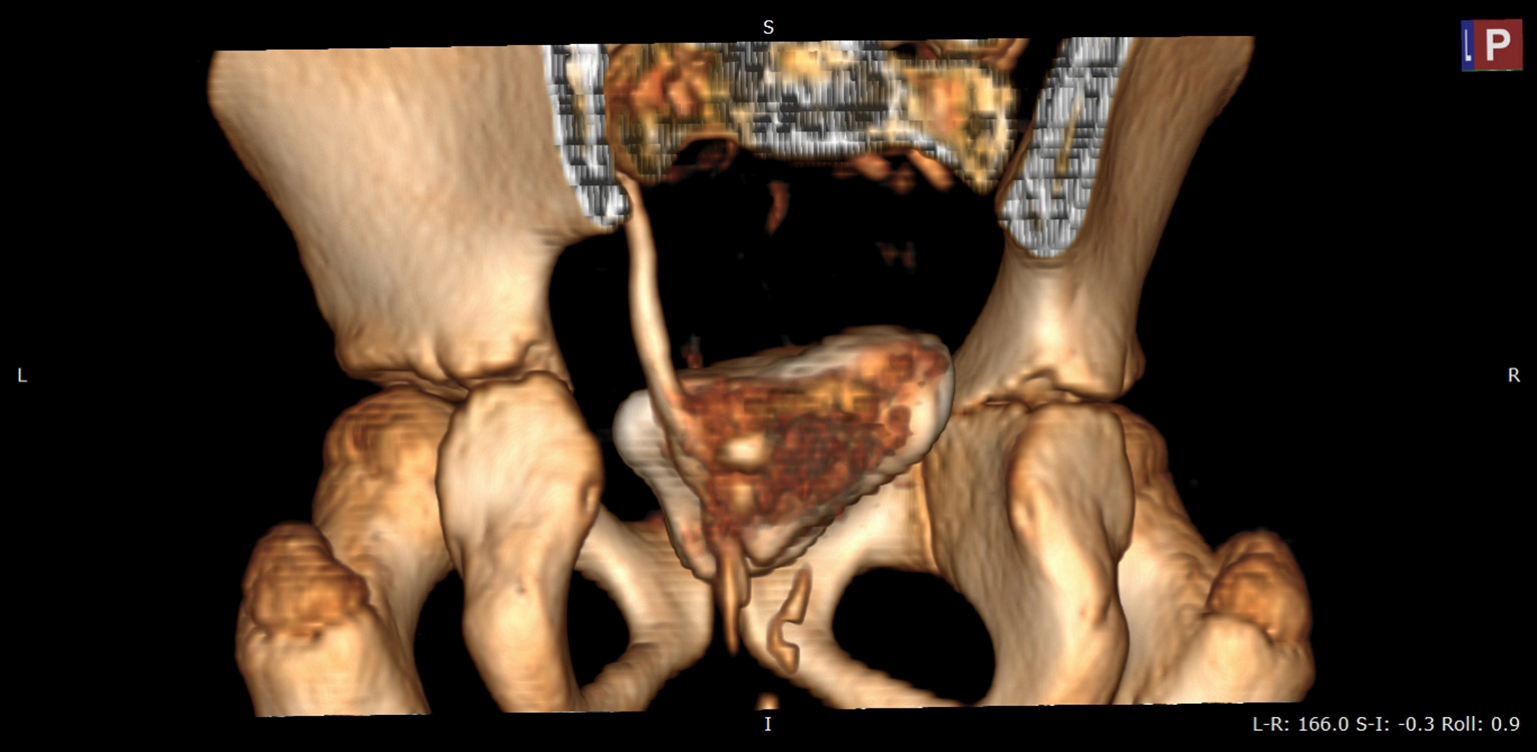
VRT (volume rendering technique) CT image, urographic sequence—posterior view after removing sacral bone—paravesical path of left ureters before insertion into vagina.

An abdominal ultrasound was performed and both kidneys had normal parenchyma and size, with a duplex-system suspicion on left side; the bladder was normal in appearance. The VCUG was repeated but no VUR was visualized. As we had a high suspicion of an ectopic ureter a contrast-enhanced computed tomography (CT) of the abdomen and pelvis was performed to visualize the entire urinary tract. The CT scan was performed with a 64 row MDCT system (Siemens Somatom Definition AS). The 3D images revealed important data about the entire collecting system and the ureters, namely a duplicated collecting system on the left side and subsequently both ureters extending from the kidney to the point of extravesical insertion into the vagina. The ectopic ureters were in very close vicinity (Figures 1–3).

In the evaluation of urinary incontinence in children of this age a differential diagnosis should be considered with the following: UTI, chronic kidney disease, diabetes, overactive bladder, ectopic ureter and vesico-vaginal fistula in case of a history of pelvic surgery.

## Discussion

This case represents a rare congenital anomaly of the genito-urinary system. An ectopic ureter is defined as a ureter that does not insert into its normal anatomical position. The occurrence of ectopic ureters is 1/2,000 in newborns and 1/2,000–4,000 in general population. Gender ratio is 2–6:1 in favor of females (1). In most cases (80–85%) the ectopic ureter is associated with a duplicated renal collecting system while in 20% a single system is found (4). The majority of cases are diagnosed during childhood, as a result of continuous urinary dribbling or recurrent UTIs. The ectopic ureter frequently occurs in association with a dysplastic upper pole renal moiety in duplex kidneys.

The patient's presentation symptoms depend on the insertion site of the ectopic ureter, and this differs between girls and boys (5). Usually, males do not present urinary incontinence because of the ureter's insertion above the external urinary sphincter, but they may present with antenatal hydronephrosis or UTIs. Like our patient, most girls present with urinary dribbling, as the insertion of the ectopic ureter will bypass the exterior urinary sphincter. Usually, affected girls have normal voiding patterns with small volume leakage or spotting incontinence. In females the most frequent sites of ectopic ureter insertion are bladder neck and upper urethra (33%), vaginal vestibule between urethra and vaginal opening (33%), vagina (25%), and less commonly the cervix or uterus (<5%) (5–7). Only girls with a ureteral insertion at or above the bladder neck and upper urethra will be continent (1).

A single collecting system with ectopic ureter is more frequently found in men, while in women the ectopic ureter is more commonly associated with a double collecting system (8).

After a literature review regarding ipsilateral or bilateral ectopic ureteral openings, only a handful of reports were found, recently Stavrinides et al reported a series of 16 isolated bilateral simplex ectopic ureter cases (9).

In our case, both ureters from a duplicated system are inserting into the vagina. This breaks Weigert-Meyer principle: usually, the upper pole ureter is ectopic and causes urinary incontinence and the lower pole ureter is inserted in the bladder. This is not the first case report that does not respect the Weigert-Meyer law. Rarely reported in the literature, “ectopic pathway” of Stephens postulates that an ectopic ureter may drain not only distally to the normal ureteric orifice (Weigert-Meyer law) but may drain medially and superiorly to it (violating Weigert-Meyer law).

Sometimes, other abnormalities like renal dysplasia and non-genitourinary anomalies (congenital heart disease, spinal cord malformations, anorectal malformations, etc) can be associated with an ectopic ureter (1, 8). In our patient no other anomaly was detected.

In assessing any child, particularly girls, with continuous urinary incontinence, accurate personal medical history and physical examination are mandatory.

In all cases imaging studies are mandatory to confirm the diagnosis (10, 11). In children, kidney ultrasound represents the initial diagnostic test, but it has some limitations and may not be so helpful. Renal ultrasound and excretory urography can almost never detect an ectopic insertion of the ureter and they do not provide enough data regarding the precise anatomy as well as in delineating the relationship between the ureter, bladder, and vagina. Therefore, contrast-enhanced computed tomography (CT) or magnetic resonance imaging urography should be the method of choice for depicting or ruling out an ectopic ureter (6). A recent report states that dimercaptosuccinic acid (DMSA) scanning is an accurate diagnostic method for dysplastic kidney detection and further CT or MRI studies do not provide additional information about ectopic ureteral insertion (12).

When ectopic ureter insertion is located in the vestibule or vagina, it causes incontinence and rarely obstruction, making its detection difficult (12). In a retrospective cohort study endoscopic examination certified the location of the ectopic ureter orifice in 70% children, and majority were in the vaginal wall except for one case in the vestibule (12).

Symptoms, renal function, patient's age and life quality will be the ones deciding the management of an ectopic ureter. In adults, conservative treatment represents an appropriate option, but does not resolve the incontinence problem and has possible long-term risks. It consists in using different types of pads according to the leakage volume (10). The best treatment in children and symptomatic patients is surgery, and it tries to resolve the incontinence, prevent further complications, preserve renal function and eliminate the UTIs. Surgical treatment consists in upper pole heminephrectomy in the non-functional duplex system or recently laparoscopic ureteral ligation (clipping) or ureteral reimplantation in case of a preserved renal function (13). Based on the reports of a new study ureteral clipping represents a faster, safer and more efficient option compared to heminefrectomy or reconstructive procedures for children (14). In 5% of patients that underwent heminephrectomy loss of renal function in the remaining ipsilateral moiety occurred, as a result of vascular injury or torsion of the renal pedicle from extensive renal mobilization (15). In case of single-system ectopic ureter with dysplastic kidney laparoscopic simple nephrectomy is recommended (12).

When there is a preserved kidney function or a functioning upper pole in duplex system kidney, the surgery technique consists on ureteroneocystostomy or distal and proximal ureteroureterostomy respectively (7). In a recent case report of a duplex system and acceptable function with an ectopic ureter draining into the uterus the approach was a laparoscopic distal ureteroureterostomy on a double J stent (7). Another surgical option in children is laparoscopic retroperitoneoscopic upper pole heminephrectomy in duplex kidney, technique that is safe for the lower pole at any age, with shorter hospital stay, better cosmetic results and lower complication rate (16).

Excellent results are reported in pediatric urology using robot-assisted laparoscopic ureteroureterostomy, that provides a safe and efficient approach to manage ectopic ureters in duplicated collecting system (15).

The surgical management of ectopic ureter depends on surgeon experience and preference, laparoscopic experience, pediatric material investments. Since the two ureters could not be separated, an “en bloc” ureteral reimplantation using the open “Cohen ureteroneocystostomy” was performed in this case. The urinary dribbling resolved postoperatively. On follow-up our patient had no UTI episode.

In conclusion, the ectopic ureter should be keep in mind in case of persistent wetting and reccurent urinary tract infections.

The exceptionality of this case, of which there are not many published, consists in the duplicated collecting system having ectopic both ureters. To the best of our knowledge, this is the first report of ipsilateral double ectopic ureter openings in children with concomitant preserved kidney function.

## Take-home message

1. A history of continuous wetting can be difficult to apprise in children.
2. A complete physical exam may be the diagnostic key for urinary incontinence.
3. The differential diagnosis of urinary incontinence in toddlers, especially girls, should include ectopic ureter.

## Ethics statement

This case report does not require a committee approval. This report does not include any identifiers of the patient to protect patient confidentiality. Written informed consent was obtained from the patient's parents with their agreement to the publication of this case report.

## Author contributions

CD and IS drafted the article and acquisition and interpretation of data; EK revised it critically for important intellectual content; CA critical revision and final approval of the version to be published.

### Conflict of interest statement

The authors declare that the research was conducted in the absence of any commercial or financial relationships that could be construed as a potential conflict of interest.
